# Systemic Chemotherapy Including Ramucirumab in Combination With Pressurized Intra-Peritoneal Aerosol Chemotherapy Is a Safe Treatment Option for Peritoneal Metastasis of Gastric Cancer

**DOI:** 10.3389/fonc.2020.610572

**Published:** 2021-04-12

**Authors:** Linda Feldbrügge, Felix Gronau, Andreas Brandl, Timo Alexander Auer, Alan Oeff, Peter Thuss-Patience, Johann Pratschke, Beate Rau

**Affiliations:** ^1^ Department of Surgery, Charité – Universitätsmedizin Berlin, corporate member of Freie Universität Berlin and Humboldt-Universität zu Berlin, Berlin, Germany; ^2^ Digestive Unit, Champalimaud Foundation, Lisbon, Portugal; ^3^ Department of Radiology, Charité – Universitätsmedizin Berlin, corporate member of Freie Universität Berlin and Humboldt-Universität zu Berlin, Berlin, Germany; ^4^ Department of Hematology, Oncology, and Tumor Immunology, Charité – Universitätsmedizin Berlin, corporate member of Freie Universität Berlin and Humboldt-Universität zu Berlin, Berlin, Germany

**Keywords:** gastric cancer, peritoneal metastasis, pressurized intraperitoneal aerosol chemotherapy, ramucirumab, peritoneal chemotherapy

## Abstract

**Background:**

Pressurized intraperitoneal aerosol chemotherapy (PIPAC) is a laparoscopic technique for local chemotherapy. It has been used for treatment of peritoneal metastasis of gastric cancer (PM GC) in combination with systemic therapy. VEGFR2 antagonist ramucirumab is a second-line therapy for GC, and has been suspected to cause wound healing disorders.

**Methods:**

This is a retrospective single center cohort study of patients with PM GC, who received PIPAC treatment in combination with systemic chemotherapy with and without ramucirumab. Data on patients’ characteristics and their perioperative courses were collected and complication rates were compared with regard to preoperative use of ramucirumab and time between last dose of systemic therapy and PIPAC treatment.

**Results:**

Fifty patients underwent 90 PIPAC treatments for PM GC in 3 years. Overall postoperative morbidity was 11% with 6% severe complications. The mean interval between systemic therapy and PIPAC was 20 days. Neither the length of interval nor the use of ramucirumab had an effect on complication rates.

**Conclusion:**

Our study suggests that addition of ramucirumab to pre-PIPAC systemic therapy, irrespective of the length of the treatment-free interval before PIPAC, does not increase the risk of postoperative complications and is therefore a safe option for treatment of PM GC.

## Introduction

Although its incidence slowly decreases in Western countries, gastric cancer (GC) still remains the third most frequent cancer related cause of death worldwide (1). In 5–20% of all GC patients, peritoneal metastasis (PM) is found at the time of diagnosis and up to 40% of stage II-III GC patients present PM during abdominal exploration (2). PM of gastric origin is associated with poor median survival rates of only 3 to 7 months without treatment (3, 4) and represents cause of death in 60% of GC patients (5).

Gastrectomy in a multimodal treatment strategy with perioperative chemotherapy is a curative treatment option for patients with locally advanced GC, including those with limited PM, in selected cases (6–8). The value of additional hyperthermic intraperitoneal chemotherapy (HIPEC) in order to prevent or delay peritoneal recurrence is controversially discussed and is currently subject of various randomized controlled trials (7, 9). However, studies suggest that a careful patient selection is fundamental, and only patients with a limited peritoneal tumor spread, defined as a peritoneal cancer index (PCI) of under 6–12, may benefit from cytoreductive surgery (CRS) including gastrectomy and peritonectomy (10, 11).

Patients with more advanced peritoneal tumor burden, who do not qualify for CRS and HIPEC, should receive systemic chemotherapy as soon as possible as it was shown to significantly increase survival, longer sustain a good quality of life, and better control symptoms (12). First-line therapy according to recent evidence should be a triple combination of fluorouracil (5-FU) plus leucovorin, oxaliplatin and docetaxel, (FLOT), whenever the patient is fit for this treatment (13), or else a double combination of oxaliplatin or cisplatin and 5-FU plus leucovorin (FLO) (14). Following the results of the RAINBOW study, anti-vascular endothelial growth factor receptor 2 (VEGFR2) antibody ramucirumab as monotherapy or in combination with paclitaxel has been approved as second line chemotherapy in Germany and other countries (12, 15).

Common systemic chemotherapy, however, is associated with severe adverse side effects such as neutropenia, diarrhea, and polyneuropathy (8, 12). Addition of ramucirumab and other angiogenesis inhibitors, such as bevacizumab, to chemotherapy has been shown to add toxicity, specifically the risk of hypertension, bleeding, and impaired wound healing (16). Besides the risk of adverse effects, according to pre-clinical studies, systemic chemotherapy might display limited penetration of the sparsely vascularized peritoneum (17–20).

Pressurized intraperitoneal aerosol chemotherapy (PIPAC) represents a novel approach to efficiently deliver chemotherapeutic agents directly to the peritoneum and thus reduce systemic side effect for patients, and potentially increase the cytotoxic efficacy. Based on the concept of therapeutic pneumoperitoneum, which was first described by Reymond in 2000 (21), PIPAC has been shown to potentially stabilize PM and even lead to histologic tumor regression in some patients (22). Others have suggested PIPAC as a potential option to reduce the Peritoneal Carcinomatosis Index (PCI) in order to facilitate CRS and HIPEC, in a neoadjuvant setting (23). Most institutions perform PIPAC in alternation with first, second, third or even fourth line intravenous chemotherapy in an effort to achieve optimal control of disease using bidirectional (systemic plus regional) cytotoxic approach (24, 25). Ramucirumab in combination with paclitaxel offers a survival benefit as second line treatment for GC patients and has therefore been reportedly used as intermittent chemotherapy regimen for PIPAC patients in some centers (25, 26).

As systemic chemotherapy, especially with addition of angiogenesis inhibition has been shown to impact wound healing (8, 27), it is common practice to maintain a certain wash out period prior to PIPAC in order to reduce the risk of systemic chemotherapy induced postoperative complications. Study protocols of current randomized clinical trials testing the addition of PIPAC to systemic chemotherapy propose an interval of 14 days (28, 29). This is also the interval that has been reportedly used by several PIPAC centers, including ours (26, 30). However, this is not in accordance with current recommendations that suggest to withhold ramucirumab at least 28 days before surgery (16). The optimal time interval between systemic chemotherapy with or without angiogenesis inhibitors and PIPAC in terms of both efficacy and safety has not been studied specifically and thus remains unclear (24).

The purpose of this study is to determine the safety of PIPAC surgery following VEGFR2 antagonist ramucirumab containing chemotherapy as a second line treatment for peritoneal metastasis of gastric cancer.

## Materials and Methods

### Data Collection

We retrospectively reviewed clinical data of all patients who underwent PIPAC for PM of GC at the Department of Surgery, Campus Charité Mitte and Campus Virchow-Klinikum, Charité Universitätsmedizin Berlin, between March 2017 and May 2020. Oncological history and prior treatments including detailed chemotherapy regimens were collected at initial consultation. Follow-up consultation was regularly performed at our institute. After PIPAC treatment, follow-up data were additionally obtained from medical oncologists or general practitioners. The last date of follow-up was the 09^th^ of April 2020. All included patients gave informed consent to collection of their personal and medical data and its use for research purposes. All data were collected, stored, and processed according to General Data Protection Regulation and local data protection laws. The study was conducted in accord with the ethical standards of the Helsinki Declaration of 1975.

### Clinical Parameters and Classifications

The preoperative general status of our patients was assessed using the American Society for Anesthesiologists physical status classification (ASA) and the Eastern Cooperative Oncology Group performance status (ECOG). Postoperative complications were categorized according to the Clavien-Dindo classification (31). Standard laboratory tests were performed pre- and postoperatively, including blood count and serum concentrations of creatinine and transaminases. Surgical site infections (SSI) were classified according to CDC (center for disease control) classification as superficial incisional SSI (I), deep incisional SSI (II), or organ/space SSI (III). Acute kidney injury network (AKIN) criteria were used for the classification of post-PIPAC acute kidney injury, defining acute kidney injury as increase of baseline serum creatinine by 1.5 fold or ≥0.3 mg/dl (32). Nodal status was either determined by a radiologist using preoperative computed tomography (CT) scans or, in case of patients who underwent gastrectomy previously, by the staff pathologist as part of the TNM tumor classification.

### Indication for PIPAC Treatment

The indication for every PIPAC procedure was discussed and confirmed individually by our multidisciplinary tumor board specialized on peritoneal malignancies. Patients were offered PIPAC procedure if they had histologically proven gastric cancer with suspected or verified PM, no other distant metastasis (except for Krukenberg tumors of the ovary), had an ECOG performance status of 2 or better, had completed at least one line of systemic chemotherapy without disease progression and were not considered for CRS and HIPEC (6). Informed consent was obtained from all patients before surgery, and included comprehensive information about the pending validation of the method in controlled clinical studies as well as alternative treatments such as systemic (second to forth line) chemotherapy alone.

### Pre- and Post-PIPAC Chemotherapy

The pre- and post-PIPAC chemotherapeutic regimen was individually decided by the treating medical oncologists, in concert with recommendations of our multidisciplinary tumor board, and based on co-morbidities, adverse side effects, previous chemotherapeutic regimens, and possible inclusion in clinical studies. We proposed three intermittent PIPAC treatments with a 2-week interval between applications of intravenous and intraperitoneal chemotherapy. Between two PIPAC surgeries, we recommended two cycles of systemic therapy (28, 29).

### PIPAC Procedure

PIPAC was performed by a small team of specialized surgeons. The standard operating procedure that we established and followed at our center was based on publications by other institutions (33–35). Drugs administered were cisplatin at a dosage of 7.5 mg/m^2^ and doxorubicin at 1.5 mg/m^2^, each dissolved in 150 ml NaCl. In brief, diagnostic laparoscopy was performed after introduction of two 12 mm trocars. Ascites, if present, was extracted for cytological analysis, and peritoneal tissue samples for histopathological examination were resected from PM. The PCI was assessed according to Jacquet and Sugarbaker (36), and documented by a short video for future comparison. Then the nebulizer (Capnopen™, Capnomed GmbH, Villingendorf, Germany) was introduced and connected with an angiographic injector (Medrad Arterion Mark 7, Leverkusen, Germany). After the staff left the operating room the aerosol was commenced with a maximum pressure of 200 psi and a flow rate of 0.5 ml/min.

The patients were mobilized and allowed to eat and drink on the day of surgery. Analgesics as well as antiemetics were prescribed routinely as follows: for analgesia paracetamol or metamizole (1 g every 8 or 6 h, respectively, intravenously), if necessary complemented by piritramide for acute postoperative pain (7.5 mg subcutaneously) and oxycodone/sustained release (10 mg every 12 h orally) for prolonged or pre-existing pain; for antiemesis ondansetron (4 mg every 6 h intravenously or orally), if necessary complemented by droperidol (1.25 mg intravenously) on demand. In absence of complications, patients were routinely discharged from the hospital on the second or third postoperative day.

### Statistics

Microsoft Excel version 2006 and IBM SPSS 26^th^ Edition (IBM, Armonk, NY, USA) were used for initial data collection and statistical analysis, respectively. Continuous variables were expressed as median (range), and compared with Mann-Whitney U-test. The Shapiro-Wilk test and the Levene test were used to test for normal distribution and homogeneity of variance, respectively. The frequencies of categorical variables were compared using Fisher’s exact test. Binary logistic regression analysis was used to test the effect of a continuous variable on a categorical dependent variable. A value of p < 0.05 was considered significant.

## Results

### Number of Sequential PIPAC Treatments and Reasons for Discontinuation

Between March of 2017 and May of 2020, 50 patients with PM GC received at least one PIPAC at our center, amounting to a total of 90 PIPAC treatments. Half of the 50 patients who received one PIPAC treatment underwent a second PIPAC and 13 patients (26%) completed three sequential treatments. Two patients (4%) had four PIPACs. Among the 37 patients who did not complete the intended three treatment cycles, but quit after one or after two PIPACs, the most common reason was cancer progress. Other reasons were preference of patient or treating medical oncologist or difficult access due to peritoneal adhesions, as determined in the first or second treatment. In five cases (10%), patients were allocated to CRS and HIPEC subsequent to PIPAC treatment. Reasons for discontinuation of PIPAC treatment are displayed in **Table 1**.

**Table 1:** Reasons for completing less than three cycles of PIPAC treatments.

Reason for discontinuation	No. of patients (%) *(n = 37)*	
Tumor progress/tumor complications	17	(45)
Preference of patient/medical oncologist	7	(19)
Subsequent CRS+HIPEC	5	(14)
Difficult access (adhesions)	4	(11)
Death	4	(11)

### Clinical Baseline and Oncological Characteristics

Clinical baseline characteristics of our patients and details of their oncological history are summarized in **Table 2**. Patients were a median 58 (31–76) years old at the time of the first PIPAC, slightly more likely to be male (n = 28, 55%), and the majority had an impaired general physical status according to ASA classification (n = 29, 58% ASA 3). On the ECOG scale for patient performance, the majority of patients (n = 41, 82%) had a good status of 0 or 1.

**Table 2:** Patient cohort: baseline and oncological characteristics.

		No. of patients (%)*(n = 50)*	
Age (years) at time of 1^st^ PIPAC	<40	5	(10%)
	40–50	11	(22%)
	50–60	12	(24%)
	60–70	18	(36%)
	>70	4	(8%)
Sex (female)		22	(45%)
ASA	1	2	(4%)
	2	19	(38%)
	3	29	(58%)
ECOG	0	20	(40%)
	1	21	(42%)
	2	9	(18%)
Histological type (Laurén)	intestinal type	4	(8%)
	diffuse type	44	(88%)
	mixed type	1	(2%)
	missing data	1	(2%)
Presence of signet cells		35	(70%)
Nodal status	N0	15	(30%)
	N1	7	(14%)
	N2	11	(22%)
	N3	17	(34%)
Synchronous PM		30	(60%)
Metachronous PM		20	(40%)
Metastasis to the ovary		4	(8%)
Gastrectomy	gastrectomy before PIPAC	23	(46%)
	curative intent*	17	(34%)
	with CRS +/− HIPEC^†^	5	(10%)
	palliative	1	(2%)
	no gastrectomy before PIPAC	27	(54%)
HIPEC without CRS (pre-PIPAC)		3	(6%)
PCI (median, min-max)		19	(1–39)

ASA, American Society for Anesthesiologists physical status; ECOG, Eastern Cooperative Oncology Group performance status; PCI, peritoneal cancer index; CRS, cytoreductive surgery; HIPEC, hyperthermic intraperitoneal chemotherapy; PIPAC, pressurized intraperitoneal aerosol chemotherapy; PM, peritoneal metastasis. *no PM at diagnosis; ^†^CRS +/− HIPEC as individualized, curative treatment for synchronous, limited PM.

With respect to the histological type of gastric cancer, our cohort was relatively homogeneous: 43 patients (88%) were identified as diffuse type according to the Laurén classification; for 35 of them (70% of all), the tumor contained signet ring cells. Four patients (8%) presented ovarian metastases. While the majority of patients (n = 35, 70%) were reportedly nodal positive, 15 patients (30%) were declared as N0, either by preoperative CT scan or by the pathologist after initial gastrectomy. At the time of the first PIPAC treatment, median PCI was 19, but showed substantial variation (range 1–39).

### History of Previous Gastrectomy, CRS, and HIPEC

In our cohort, 30 patients (60%) had synchronous PM while 20 (40%) developed PM after initial diagnosis of GC. Of the 20 cases with metachronous PM, 17 patients previously underwent gastrectomy with curative intent. The other three patients were diagnosed with PM after neoadjuvant chemotherapy, and therefore did not receive the intended gastrectomy. Of the 30 patients with synchronous PM, six patients also underwent gastrectomy before first PIPAC: five gastrectomies were performed in combination with CRS with or without HIPEC as an individualized treatment concept for limited, synchronous PM, and one patient received palliative gastrectomy for tumor bleeding. Three patients had received HIPEC without CRS prior to first PIPAC treatment.

**Figure 1:**
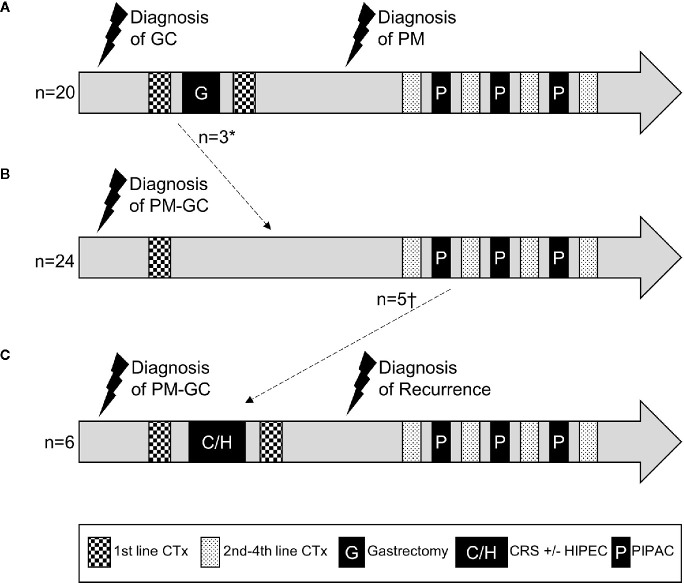
Schematic treatment courses of patients with gastric cancer and metachronous **(A)** or synchronous **(B, C)** peritoneal metastasis. Numbers and regimens of chemotherapies and numbers of PIPACs varied between patients and are depicted symbolically. *diagnosis of PM at the time of intended curative gastrectomy, which was then aborted. †patients who received CRS+/**−**HIPEC after first or second PIPAC treatment. GC, gastric cancer; PM, peritoneal metastasis; CTx, chemotherapy; CRS, cytoreductive surgery; HIPEC, hyperthermic intraperitoneal chemotherapy; PIPAC, pressurized intraperitoneal aerosol chemotherapy.

**Figure 1** visualizes these different clinical and treatment courses of our GC patients with PM depending on the order of diagnosis. Numbers of chemotherapy and PIPAC cycles varied widely and are depicted symbolically.

### Length of Stay and Postoperative Complication Rates

The median duration of a PIPAC procedure was 75 min (37–116). The length of surgery did not differ between the first or any of the subsequent PIPACs. Patients stayed a median of three nights (2–43) in the hospital before discharge. The length of stay (LOS) did not change with the number of PIPAC treatments. Out of 90 performed PIPACs, patients only stayed longer than five nights in five cases (6%), four of which were due to additional treatments of their underlying tumors: endoscopy for bleeding of the primary tumor (in two cases) and pleurodesis for pleural effusion (2^nd^ and 3^rd^ PIPAC treatments of the same patient). The fifth case was due to bowel perforation which caused several re-operations and an extended hospital stay of 43 nights.

Out of 90 PIPACs, there was one case (1%) of stage 1 acute kidney injury as defined by the AKIN criteria. This male patient had an increased postoperative serum creatinine level that was 1.6-fold his preoperative value, but that was still in the normal range (1.01 mg/dl) and decreased to baseline levels under conservative treatment. There was no significant change in serum levels of alanine aminotransferase (ALT) before and after PIPAC treatment in any case, indicating no relevant hepatic parenchymal injury.

Overall postoperative morbidity was 11% (10 cases), and severe complications as assessed by Clavien-Dindo classification of grade three or more, were reported in five cases (6%). Four patients were re-admitted to the hospital after initial discharge due to complications, and four patients underwent re-operation. All postoperative complications, their management, and re-admissions are detailed in **Table 3**.

**Table 3:** Postoperative complications and re-admissions after PIPAC treatments.

Severity	No. of cases (%) *(n = 90)*	Type of complication	Treatment	Reason for re-admission
**1**	5 (6%)	Nausea (n = 2) Surgical Site Infection Grade I (n = 2) Acute kidney injury Stage 1 (n = 1)	Conservative management Conservative management Conservative management	n = 1
**2**	0			
**3a**	1 (1%)	Surgical Site Infection Grade III (n = 1)	Interventional drainage	n = 1
**3b**	4 (4%)	Small bowel perforation (n = 1) Surgical Site Infection Grade II (n = 1) Trocar site hernia (n = 1) Urinary infection + acute abdomen (n = 1)	Re-operation (laparotomy + ileostomy) Re-operation (surgical debridement and re-closure of fascia) Re-operation (hernia repair) Re-operation (diagnostic laparoscopy, for acute abdomen; no pathological finding. Most likely cause was urinary infection.)	n = 1 n = 1
**4-5**	0			

Severity classification according to Clavien-Dindo. Surgical Site Infection grades according to CDC classification.

### History of Chemotherapies

All patients in our cohort underwent at least one line of chemotherapy before their first PIPAC treatment. However, as our cohort comprises both patients with metachronous PM and patients with synchronous PM, our patients’ histories display a wide range of chemotherapy regimens that vary in medication, length, number of treatments, and treatment intention (curative *vs.* palliative). To examine the impact of prior systemic chemotherapy with or without ramucirumab on the safety of PIPAC treatment, we have analyzed the last systemic therapy patients received before PIPAC procedure, which is detailed in **Table 4**. For this analysis, we did not consider any therapies that were administered more than 6 weeks (42 days) prior to PIPAC. This selection excluded 13 cases (14%), thus leaving 77 PIPACs (86%) to be analyzed for pre-PIPAC systemic therapies and their relation with postoperative complications.

**Table 4:** History of chemotherapy regimens preceding PIPAC treatment.

	No. of cases (%) (n = 77)^*^	
Paclitaxel + ramucirumab	30	(39%)
FLOT	21	(27%)
FLO	10	(13%)
FOLFIRI	5	(7%)
FOLFIRI + ramucirumab	2	(3%)
EOX/ECF/ECX	2	(3%)
Capecitabine/5-FU + FA	4	(5%)
5-FU + ramucirumab	3	(4%)
any chemotherapy without ramucirumab	42	(54.5%)
any chemotherapy with ramucirumab	35	(45.5%)

*Cases with more than 42 days interval since last chemotherapy dose (n = 13) were excluded. FLO, 5-fluorouracil (5-FU) + leucovorin + oxaliplatin; FLOT, FLO+ taxane (docetaxel); FLT, 5-FU + leucovorin + taxane; FLC, 5-FU + leucovorin + cisplatin; Xelox, oxapliplatin + capecitabine; EOX, epirubicin + oxaliplatin + capecitabine; ECX, epirubicin + cisplatin + capecitabine; ECF, epirubicin + cisplatin + 5-FU; FOLFIRI, 5- FU + leucovorin + irinotecan.

The last treatment before PIPAC included ramucirumab in 35 cases (45%), most commonly in combination with paclitaxel. Most patients who did not receive ramucirumab, were treated with a double (FLO) or triple (FLOT) combination therapy including oxaliplatin.

The median number of cycles of the last chemotherapy regimen that patients received prior to PIPAC surgery was two (1–10). The number depended significantly on the number of PIPAC surgery: patients received a median of four cycles (1–10) before their first PIPAC treatment, while the median number of cycles between PIPAC treatments was two (1–2; p < 0.001).

### Addition of Ramucirumab Does Not Increase Postoperative Complication Rates

Out of the 35 PIPACs that were performed within 42 days after the administration of ramucirumab, three cases had postoperative complications: one SSI, one bowel perforation, and one case of early postoperative urinary infection that led to diagnostic laparoscopy for acute abdomen. Comparing patients who had received ramucirumab before PIPAC treatment with those who had not, there was no significant difference in overall postoperative morbidity, severe complication rate, or length of stay, as shown in **Table 5**.

**Table 5:** Effect of ramucirumab (RAM) addition to pre-PIPAC chemotherapy (CTx) on postoperative complication rates and length of stay (LOS).

	CTx - RAM	CTx + RAM	
	*(n = 42)*	*(n = 35)*	*p-value*
Overall morbidity	4 (10%)	3 (9%)	1.000
Severe complication	2 (5%)	3 (9%)	0.654
LOS (median, min-max)	3 (2-6)	3 (2-43)	0.211

Severe complications are classified as Clavien-Dindo ≥3a.

### Shorter Interval Between Systemic Chemotherapy and PIPAC, Irrespective of Addition of Ramucirumab, Does Not Increase Postoperative Complication Rates

When scheduling procedures, we recommended a pause of at least 14 days between PIPAC treatment and chemotherapy and *vice versa*. In 13 cases, patients of our cohort had received chemotherapy more than 42 days before a PIPAC treatment (most commonly before their first PIPAC). These cases excluded, there was a median of 20 days (7–41) interval between last day of chemotherapy and PIPAC treatment. Interestingly, patients treated with an addition of ramucirumab had a significantly shorter interval between systemic treatment and PIPAC procedure than patients without ramucirumab (18 days (7–38) *vs* 21 days (14–41), p = 0.02).

Patients who received systemic chemotherapy less than 14, 21, or 28 days before PIPAC surgery, respectively, were not more likely to develop postoperative complications. This was true irrespective of the addition of ramucirumab to systemic chemotherapy (**Table 6**). Days between last dose of systemic therapy and PIPAC also did not predict postoperative complications in a logistic regression model (odds ratio 1.038; 95% confidence interval 0.941–1.144; p = 0.458).

**Table 6:** Effect of interval between systemic chemotherapy with or without ramucirumab (+ RAM or – RAM, respectively) and PIPAC on postoperative complication rates.

Interval (days)	≤14		15–21		22–28		>28	
CTx – or + RAM	− RAM	+ RAM	− RAM	+ RAM	− RAM	+ RAM	− RAM	+ RAM
No. of patients	*n = 2*	*n = 9*	*n = 20*	*n = 17*	*n = 11*	*n = 5*	*n = 8*	*n = 4*
Overall morbidity	1	0	1	1	0	2	2	0
Severe complications	1	0	1	1	0	2	0	0

Severe complications are classified as Clavien-Dindo ≥3a.

## Discussion

PIPAC is a novel, innovative strategy for intraperitoneal application of chemotherapy for the treatment of advanced stages of PM GC. One potential benefit of adding intermittent local anti-cancer therapy is to bridge longer breaks of systemic chemotherapy and thus permit a prolonged overall duration of treatment without adding to systemic toxicity. While large randomized controlled trials are still lacking, several smaller observational studies suggest that PIPAC may promote histological tumor regression, attenuate development of ascites, improve survival, and might facilitate subsequent CRS and HIPEC (23, 26, 37).

According to current guidelines, systemic chemotherapy is essential for the treatment of patients with PM GC, and as clinical trials have not yet proven the efficacy of PIPAC treatment, any delay in the administration of systemic chemotherapy has to be averted. It is therefore crucial to assess possible effects of preoperative systemic therapies on postoperative complications and determine the optimal time interval between systemic chemotherapy and PIPAC. Several studies have demonstrated that PIPAC is a safe procedure with low postoperative complication rates (22, 25, 30, 38). However, most studies comprise heterogeneous groups with different tumor entities and therefore different preceding systemic therapies, and do not provide details on preoperative therapies and time intervals between systemic treatment and PIPAC.

We here report a single-institution cohort of patients that homogeneously present with PM GC and receive between one and four PIPAC treatments, with the aim of analyzing their postoperative complications in relation to their preoperative systemic treatments. We observe an overall postoperative morbidity of 11% in PM GC patients after PIPAC with severe complications in 6%, which is in keeping with other studies (39, 40). Almost half (45%) of systemic therapies obtained within 6 weeks before PIPAC treatments included ramucirumab. In fact, the median interval between ramucirumab-containing systemic treatment and PIPAC was 18 days, and thus 3 days shorter than that between other chemotherapy and PIPAC. Nevertheless, we did not observe any increase in complication rates or LOS after prior treatment with ramucirumab, when compared with patients who received ramucirumab-free chemotherapy. Furthermore, regardless of the composition of chemotherapy, outcomes did not depend on the interval between last dose of systemic therapy and PIPAC surgery. To date, no study has been published about the optimal interval between systemic therapy and PIPAC surgery, especially with a focus on potential adverse effects of ramucirumab.

Angiogenesis inhibitors that target the formation of aberrant, pathological vessels by a tumor, have been shown to be effective anti-cancerous agents. Especially anti-VEGF-A antibody bevacizumab has become an established component of systemic anti-cancer therapy for tumors of the brain, lung, breast, and colorectal cancer (41, 42). However, bevacizumab was shown to impair wound healing as demonstrated in the context of colorectal liver metastasis, treated surgically after neoadjuvant chemotherapy including bevacizumab. This has led to the official recommendation of delaying surgery for 6 weeks after treatment with bevacizumab (43–45). Eveno et al. could show that neoadjuvant bevacizumab treatment significantly increases incidence of major perioperative complications, wound healing disorders, intraabdominal abscess formation and leads to longer hospitalization in colorectal cancer patients undergoing CRS and HIPEC, although a 6-week wash-out period had been upheld (46). On the other hand, when focusing on patients with gastro-esophageal cancers, Okines et al. found that bevacizumab did not increase perioperative complications including bleeding and wound healing disorders with 5 to 6 weeks between bevacizumab and surgery (47).

Anti-VEGFR2 antibody ramucirumab was more recently added to the portfolio of angiogenesis inhibitors for solid tumor therapy. Its first FDA approval was passed in 2014 for second line treatment of advanced gastric cancer (15). Like bevacizumab, ramucirumab has been associated with an increased rate of wound healing disorders. However, this correlation is only based on a total of 18 cases of wound-healing complications (14 after ramucirumab, 4 in control groups) in a meta-analysis comprising 4,996 patients (27). Its effects on perioperative complications, more specifically after minimally-invasive surgery such as PIPAC have not been studied to date.

Recently, Siebert et al. described a cohort of 26 patients who underwent PIPAC procedures after receiving bevacizumab and compared their complication rates with 108 patients who were treated without bevacizumab. Interestingly, in this study, bevacizumab treatment was not associated with increased perioperative morbidity (48). While this is an important observation, being the first study that explicitly examines PIPAC complications after angiogenesis inhibition, it has to be noted that this study cohort was very heterogeneous, and the bevacizumab group did not include any patients with gastric cancer. Furthermore, prior chemotherapies and the time intervals between systemic therapy and PIPAC were not presented in detail.

While our patient demographics and oncological classifications are in accordance with other reports about advanced GC, some characteristics of our cohort should be pointed out for precise interpretation of the results. First, as our patient cohort comprises patients with both synchronous and metachronous peritoneal metastasis, there are substantial differences in their individual medical history, such as prior gastrectomy, duration and number of cycles of prior chemotherapy. These factors are likely to influence survival, which is why we have not intended, in this cohort, to analyze survival. However, the influence of ramucirumab or other chemotherapies on short-term postoperative outcome after PIPAC is unlikely to be impacted by long-term differences in medical history, especially since general health, as measured with ASA and ECOG scores, was very homogeneous in our group. Only a randomized controlled trial could establish with certainty that oncological history, prior surgeries, or other, unknown factors do not have significant influence on chemotherapy induced perioperative morbidity. The total number of complications in our cohort is too low for a meaningful multivariate analysis, that could otherwise strengthen our results in this regard.

In conclusion, despite these limitations due to the nature of our retrospective study, we here present the first study that analyzes safety of PIPAC for peritoneal metastasis of gastric cancer in combination with systemic therapy including ramucirumab. Our data suggest that addition of ramucirumab to systemic therapy, even with a treatment-free interval as short as 2 weeks before PIPAC surgery, does not increase the risk of postoperative complications. Still, randomized controlled clinical trials are urgently needed to confirm efficacy of PIPAC treatment and ascertain optimal combination and timing of inductive and intermittent systemic chemotherapy.

## Data Availability Statement

The datasets presented in this article are not readily available because Patients clinical data. Requests to access the datasets should be directed to beate.rau@charite.de.

## Ethics Statement

The studies involving human participants were reviewed and approved by Charité Universitätsmedizin Berlin, Ethikkommission. The patients/participants provided their written informed consent to participate in this study.

## Author Contributions

LF designed the study, acquired the data, analyzed the data, and wrote the manuscript. PT-P and BR designed the study, wrote and critically revised the manuscript. AB designed the study, acquired and analyzed the data, and wrote the manuscript. FG, TAA, and AO acquired and analyzed the data and wrote the manuscript. JP supervised and designed the study and critically reviewed the manuscript. All authors contributed to the article and approved the submitted versions.

## Funding

LF is supported by DFG (Deutsche Forschungsgemeinschaft) FE1434.2-1.

## Conflict of Interest

PT-P is compensated for being on the Advisory Boards of Roche, Bristol-Myers Squibb, Merck Sharp & Dohme, Merck Serono, Lilly and Servier.

The remaining authors declare that the research was conducted in the absence of any commercial or financial relationships that could be construed as a potential conflict of interest.
